# Correlation between executive function and quantitative EEG in patients with anxiety by the Research Domain Criteria (RDoC) framework

**DOI:** 10.1038/s41598-020-75626-0

**Published:** 2020-10-29

**Authors:** Su Hyun Bong, Tae Young Choi, Kyoung Min Kim, Jaewon Lee, Jun Won Kim

**Affiliations:** 1 Department of Psychiatry, Catholic University of Daegu School of Medicine, 33, Duryugongwon-ro 17-gil, Nam-gu, Daegu, 42471 Republic of Korea; 2 Department of Psychiatry, Dankook University Hospital, Cheonan, Republic of Korea; 3 Department of Psychiatry, Easybrain Center, Seoul, Republic of Korea

**Keywords:** Emotion, Neuronal physiology

## Abstract

The Research Domain Criteria (RDoC) project was proposed by the National Institute of Mental Health in 2010 to create a new diagnostic system including symptoms and data from genetics, neuroscience, physiology, and self-reports. The purpose of this study was to determine the link between anxiety and executive functions through quantitative electroencephalography (qEEG) based on the RDoC system. Nineteen-channel EEGs were recorded at the psychiatric clinic from 41 patients with symptoms of anxiety. The EEG power spectra were analysed. The Executive Intelligence Test (EXIT) including the K-WAIS-IV, Stroop, controlled oral word association, and the design fluency tests were performed. A partial, inversed, and significant association was observed between executive intelligence quotient (EIQ) and the absolute delta power in the central region. Similarly, a partial, inversed, and significant association was observed between design fluency and the absolute delta power in the left parietal area. Our findings suggest that the increase in delta power in the central region and left P3 was negatively correlated with the decrease in executive function. It is expected that the absolute delta power plays a specific role in the task-negative default mode network in the relationship between anxiety and executive function.

## Introduction

The Diagnostic and Statistical Manual of Mental Disorders Fifth Edition (DSM-5) is a representative classification system of mental disorders that are defined by symptoms and behaviours that are either reported by patients or observed by clinicians^1^. The DSM-5 has greatly contributed to the improvement of reliability in the clinical and psychiatric research field. However, the limitation that individuality was lost in the evaluation, treatment, and prognosis, as symptoms of various patients are diagnosed as a single disorder was evident. Moreover, another limitation refers to the absence of contemporary neuroscientific technology for diagnostic purposes, contradicting other fields including neurosurgery, rehabilitation medicine, etc^2^. As a result, the Research Domain Criteria (RDoC) project was proposed by the United States National Institute of Mental Health in 2010 to create a new diagnostic system that not only includes symptoms, but also data from genetics, neuroscience, physiology, and self-reports. The RDoC is not a classification system for clinical diagnosis; it is a system for psychiatric disease research and is classified into five research areas: positive valence; negative valence; cognitive function; social process; and arousal/modulation. Research is actively conducted worldwide^3^. More particularly, regarding anxiety, there is an RDoC anxiety depression project (RAD project), and the brain-based constructs and symptoms are linked and explained through brain imaging^4^.

Anxiety, which is one of the most common psychopathologies, is a complex emotional response with both physiological and psychological symptoms. It is a phenomenon in which unpleasant psychological conditions, such as subjective tension and irritability, are felt by the patient and are accompanied by various physiological reactions of the autonomic nervous system, such as tachycardia, tremor, and dizziness^1^. Previously, clinicians had to rely on the patients' self-reports and measures to assess symptoms of anxiety. However, there have been increasing reports that electroencephalography (EEG) may help the diagnosis of various mental disorders that show brain dysfunction. Specifically, quantitative EEG (qEEG), a quantitative analysis of brain waves, is a technique that is currently widely used in a clinical, cognitive psychological, or engineering manner. Its advantages include that it is a non-invasive method that does not involve radiation exposure, it has a high time resolution, and excellent accessibility due to a low inspection cost^5^. When qEEG was measured in most patients with an anxiety disorder, both an alpha wave asymmetry^6^ and an increase in beta waves^7^ were commonly reported. These phenomena are interpreted as the underlying instability of cortical arousal and hyperactivation of the frontal cortex^8^. However, since symptoms persist in various ways besides anxiety, there is no consistent EEG result^9^. Therefore, studies measuring the reliability of quantitative EEG as a diagnostic tool in mental disorders are still in progress.

The neuropsychological function of the frontal lobe, which is termed as executive function, includes cognitive flexibility, creativity, planning, abstract thinking, insight, spontaneity, deterrence, emotion, personality, and social behaviour. Executive functions govern higher behaviour; they modulate it by coordinating information coming from other association areas. It is known that anxious people often experience cognitive function and concentration deterioration and present related abnormalities in other brain areas^10^. Electrophysiological tests, including cognitive function and brain magnetic resonance imaging, and EEG in patients with anxiety disorders have confirmed overall frontal lobe processes, especially regarding the prefrontal lobe, that is responsible for cognitive function execution through top-down inhibitory control deficits^11^. Here, unlike previous studies that were limited to a single disease as a source of interest, anxiety symptoms were defined by the RDoC system to assess the relationship between qEEG and executive functions.

## Results

### Demographic and clinical data

Forty-one patients (11 males and 30 females) volunteered to participate after being informed of the study’s purpose and methodology. The participants’ mean age was 53.00 ± 13.56 and 52.53 ± 11.09 for males and females, respectively. The demographic and clinical data are summarised in Table 1.

**Table 1:** Demographic data and cognitive function of subjects.

Mean ± SD	All subjects (n = 41)
**Gender, n (%)**	
Male	11 (26.8)
Female	30 (73.2)
**Age**	
Male	53.00 ± 13.56
Female	52.53 ± 11.09
BDI	31.1 ± 12.52
STAI (state)	59.63 ± 11.74
STAI (trait)	59.54 ± 10.86
**Executive function**	
EIQ	109.85 ± 13.98
**Stroop test**	
Simple trial	13.12 ± 2.69
Midterm trial	12.63 ± 3.78
Interference trial	13.32 ± 3.57
Verbal fluency	13.07 ± 3.25
Design fluency	11.83 ± 2.57

*SD* standard deviation, *BDI* Beck Depression Inventory, *STAI* Korean version of State-Trait Anxiety Inventory, *EIQ* Executive Intelligence Quotient.

### Correlation analysis

**Figure 1:**
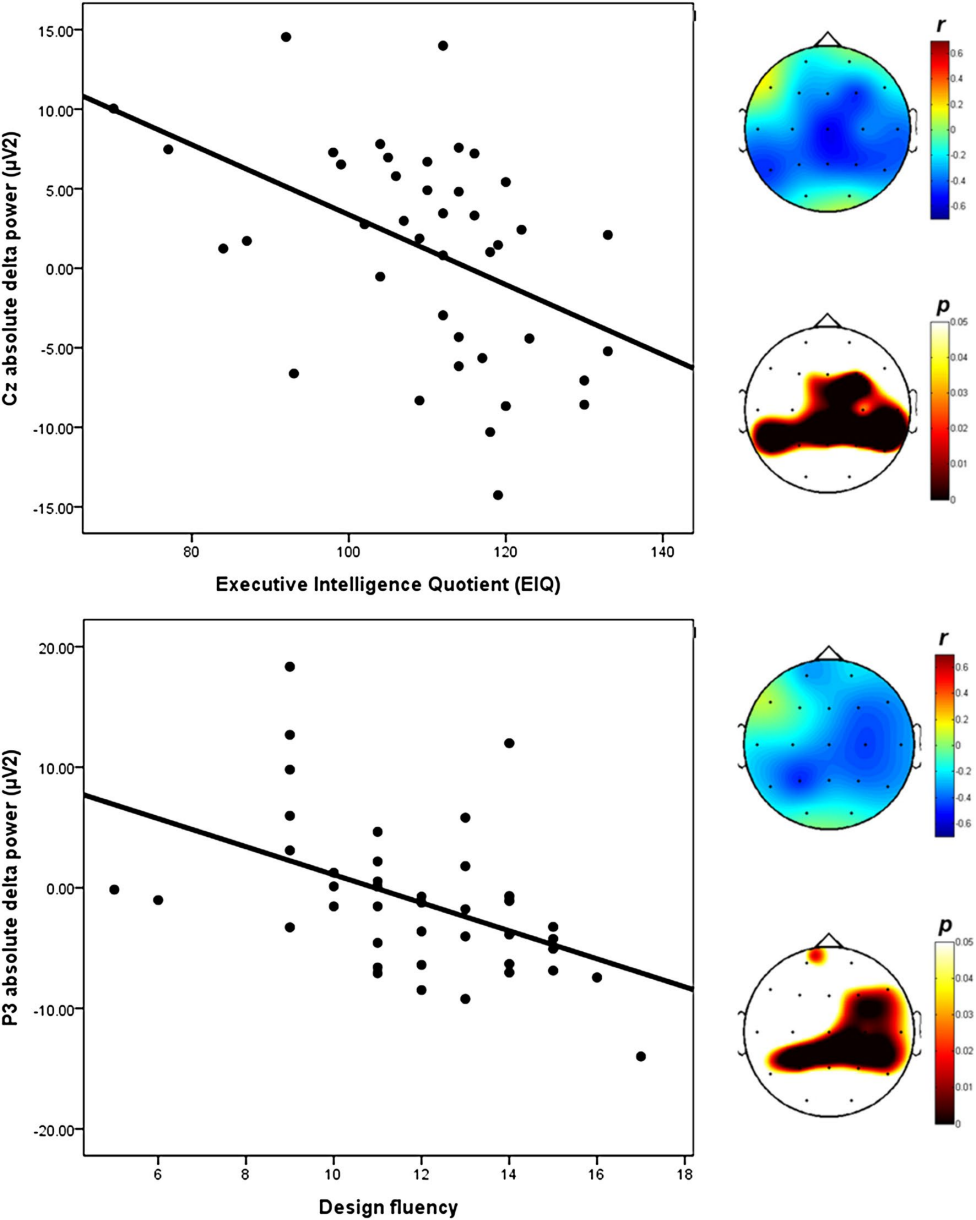
Topographical representations of the Pearson’s partial correlation, corrected for age, gender and BDI, between the absolute delta power and the EIQ and design fluency scores. On the top is the topography of the Pearson’s partial correlation coefficients, and on the bottom is the topography of the associated *p* values. r, Pearson’s partial correlation coefficient; *p*, *p* value; BDI, Beck Depression Inventory; EIQ, Executive Intelligence Quotient.

Pearson’s partial correlation analyses were performed on the executive function and EEG recordings. We found that the EXIT score was negatively correlated with the absolute delta power. Two significant findings were revealed after applying the Bonferroni correction (*p* < 0.0026). First, a partial, inversed, significant association was observed between the Executive Intelligence Quotient (EIQ) and the absolute delta power in the central region (Cz, r = -0.553, *p* < 0.0001). Similarly, a partial, inversed, significant association was observed between design fluency and the absolute delta power in the left P3 (r = -0.482, *p* = 0.0021). The partial correlation analysis between the absolute theta, alpha, beta powers, and the executive function demonstrated no significant relationship. The results of Pearson’s partial correlation analysis that controlled for age, gender, and BDI between the executive function and EEG analyses are presented in Table 2. The scatter plots and topographical features of the partial correlation are also presented in Fig. 1. In addition, Pearson's partial correlation analyses were conducted in the same way to analyse the relationship between 1) anxiety and EEG analyses, and 2) anxiety and cognitive function, though no significant correlations were identified. The results are tabulated in Supplementary Table S1, S2, and S3 Online.

**Table 2:** The results of the Pearson’s partial correlation analysis (corrected for age, gender and BDI) between cognitive function and EEG analysis.

Absolute delta	EIQ	Stroop			Verbal	Design
		Simple	Midterm	Interference		
**Fp1**						
*R*	− 0.186	0.081	0.166	0.005	− 0.060	− 0.311
*P*_*Bon*_	0.2637	0.6274	0.3195	0.9758	0.7184	0.0577
**Fp2**						
*R*	− 0.088	0.073	0.197	0.197	0.029	− 0.232
*P*_*Bon*_	0.6013	0.6643	0.2362	0.2359	0.8616	0.1616
**F7**						
*R*	0.102	− 0.100	− 0.003	0.090	− 0.098	0.058
*P*_*Bon*_	0.5422	0.5510	0.9863	0.5902	0.5590	0.7294
**F3**						
*R*	− 0.279	− 0.293	− 0.131	− 0.165	− 0.185	− 0.113
*P*_*Bon*_	0.0895	0.0746	0.4327	0.3236	0.2665	0.5001
**Fz**						
*R*	− 0.328	− 0.165	− 0.167	− 0.252	− 0.218	− 0.269
*P*_*Bon*_	0.0442	0.3234	0.3149	0.1269	0.1878	0.1022
**F4**						
*R*	− 0.474	− 0.305	− 0.169	− 0.286	− 0.216	− 0.394
*P*_*Bon*_	0.0027	0.0623	0.3104	0.0821	0.1922	0.0143
**F8**						
*R*	− 0.203	− 0.102	− 0.177	− 0.051	− 0.222	− 0.355
*P*_*Bon*_	0.2223	0.5441	0.2873	0.7594	0.1801	0.0289
**T7**						
*R*	− 0.268	− 0.194	− 0.212	− 0.276	− 0.229	− 0.198
*P*_*Bon*_	0.1038	0.2439	0.2013	0.0930	0.1667	0.2331
**C3**						
*R*	− 0.301	− 0.237	− 0.205	− 0.157	− 0.211	− 0.239
*P*_*Bon*_	0.0659	0.1515	0.2178	0.3456	0.2032	0.1478
**Cz**						
*R*	− 0.553*	− 0.279	− 0.302	− 0.330	− 0.350	− 0.363
*P*_*Bon*_	**0.0000**	0.0896	0.0656	0.0428	0.0312	0.0249
**C4**						
*R*	− 0.356	− 0.143	− 0.176	− 0.308	− 0.034	− 0.450
*P*_*Bon*_	0.0280	0.3929	0.2898	0.0596	0.8406	0.0046
**T8**						
*R*	− 0.397	− 0.350	− 0.241	− 0.196	− 0.390	− 0.348
*P*_*Bon*_	0.0136	0.0311	0.1449	0.2388	0.0155	0.0322
**P7**						
*R*	− 0.475	− 0.363	− 0.313	− 0.232	− 0.213	− 0.298
*P*_*Bon*_	0.0026	0.0251	0.0557	0.1618	0.1996	0.0691
**P3**						
*R*	− 0.386	− 0.306	− 0.174	− 0.175	− 0.056	− 0.482*
*P*_*Bon*_	0.0166	0.0613	0.2953	0.2936	0.7363	**0.0021**
**Pz**						
*R*	− 0.464	− 0.227	− 0.069	− 0.031	− 0.238	− 0.346
*P*_*Bon*_	0.0033	0.1701	0.6794	0.8534	0.1494	0.0334
**P4**						
*R*	− 0.467	− 0.353	− 0.275	− 0.263	− 0.389	− 0.373
*P*_*Bon*_	0.0032	0.0298	0.0943	0.1103	0.0158	0.0210
**P8**						
*R*	− 0.402	− 0.154	− 0.293	− 0.392	− 0.241	− 0.337
*P*_*Bon*_	0.0123	0.3550	0.0739	0.0148	0.1450	0.0386
**O1**						
*R*	− 0.145	− 0.026	− 0.019	− 0.217	− 0.080	− 0.117
*P*_*Bon*_	0.3858	0.8766	0.9120	0.1909	0.6350	0.4845
**O2**						
*R*	− 0.055	− 0.185	− 0.101	− 0.235	− 0.146	− 0.133
*P*_*Bon*_	0.7436	0.2654	0.5449	0.1549	0.3815	0.4248

* *p* < 0.0026(0.05/19), R means Pearson’s partial correlation coefficient; P means *p* value of Pearson’s partial correlation; P_Bon_ means the *p* value adjusted using the Bonferroni correction; BDI, Beck Depression Inventory; EEG, electroencephalogram, EIQ, Executive Intelligence Quotient.

## Discussion

In this study, we found that a decrease in executive function may be associated with an increase in absolute delta power in patients with anxiety. To the best of our knowledge, this is the first study that attempted to illustrate the link between anxiety and executive function from the perspective of the RDoC framework by use of qEEG. This study is most suitable for the potential threat (anxiety) construct of the negative valence domain among the five domains of the RDoC framework^3^. While it may be thought that the study of the relationship between executive function and anxiety is related to the various constructs of the cognitive system domain, for patients with anxiety as their main complaint, the potential threat construct may be more reasonable. In particular, we examined whether the qEEG parameters' absolute power is related to a decrease in executive function in anxiety by using qEEG; thus, it can be said that units of analysis correspond to physiology. Based on the results of this study, it seems that high absolute delta power (as ‘physiology’) both in the left parietal (P3) and central (Cz) areas is related to potential threats and poorer executive function.

In previous studies, anxiety has been largely divided into two categories: anxious arousal and anxious apprehension^12^. Anxious arousal is mainly characterised by physical tension and psychological arousal, while anxious apprehension is characterised by verbal and behavioural ruminations. In particular, anxious apprehension had been associated with problems in cognitive areas, such as negative thoughts and delayed execution functions, and EEG and functional magnetic resonance imaging (fMRI) studies have revealed a dominant left hemisphere activity^13,14^. Moreover, anxious apprehension impairs executive functions in the cascade-of-control model^15^. In previous studies using event-related potentials and fMRI, the left dorsolateral prefrontal cortex and dorsal anterior cingulate cortex were identified as brain regions that mediate anxious apprehension and impairment of executive function^16^. In patients with panic disorder, a decrease in working memory has been observed, in which gamma coherence increases during communication between the left frontal and parietal areas^17^. Due to these points, it seems that the cortex of the left hemisphere mainly played a role in mediating anxiety and executive function in the existing studies.

Conversely, we observed that the absolute delta power on the qEEG was inversely proportional to the decrease in executive function of anxious patients. Brain waves are known to be mainly generated from oscillators, and waveforms with low vibration frequencies, such as delta waves, affect a large area far from the surface^18^. In particular, delta waves are common in the deepest stage of slow-wave sleep, and its oscillator is mainly located in the brain stem^19^, thalamus^20^, cortex^21^, etc. In addition, delta waves are related to the upstate of the cortex in that the slow resting-state oscillation is related to the cortex rather than the brain stem^22^. Given that delta waves may be increased due to the lack of arousal of the brain^23^, resting-state delta wave elevations are clinically associated with a decreased function of the brain’s cortex. In particular, the activation of the P3 is typical in anxiety^24^, and executive functions such as attention control and working memory are related to P3 activation^25^. It is consistent with the results of this study that the increase in delta power in the P3 correlated with the decrease in executive function in patients with anxiety symptoms. Furthermore, this study's hypothesis is supported by the previous study’s results of anxiety reduction by inhibiting the P3 through the application of 1 Hz low frequency repetitive transcranial magnetic stimulation^26^.

Delta wave power correlated with the brain area responsible for the memory process in the DMN^27^, which is a type of resting-state network that is inhibited during task performance and activated in the brain of the resting state, and is related to low-frequency neuronal oscillation, known to affect emotion and cognition in the posterior cingulate cortex and medial prefrontal cortex^28^. We found that the executive function negatively correlated with absolute delta power in Cz and P3 in patients with anxiety. In a recent study using EEG and fMRI, it was found that Cz has connectivity with the posterior cingulate cortex, and P3 has connectivity with the lateral-parietal cortex and medial prefrontal^29^. These brain regions are known to be strongly associated with DMN, so it is presumed that an increase in delta power may have lowered executive function in patients complaining of anxiety related to abnormal DMN function. Though research using qEEG is highly effective in analysing DMN^30,31^, the use of 19 channels, which is a relatively small number, has a disadvantage that spatial resolution is relatively lower than fMRI. To overcome these limitations attempts to understand brain physiology using Simultaneous EEG-fMRI have been actively conducted recently^32–34^. Therefore, to expand the results of this study, if fMRI is combined, a wider understanding of the phenomenon will be possible. Moreover, DMN is known to be activated in the task-negative network and inhibited in the task-positive network^28^. In this study, there are limitations in interpreting the results due to the absence of control using tasks that stimulate emotions and cognitions; therefore, further study is needed.

The limitations of this study are: first, since it was conducted without a control group, it is difficult to specify this as a result that occurs only in individuals who complain of anxiety. Therefore, in future studies, there is a need to study whether an increase in absolute delta power in anxiety and executive function appears only when complaining of anxiety using a healthy control. Second, this study’s sample size was relatively small (n = 41), of which only 11 were male. A small number of participants may be correlated with this study’s results showing negative findings regarding other indicators except for delta waves; therefore, future research should include more participants to obtain more accurate and specific results. Third, as the results of the study described above, DMN seems to play a role in the relationship between anxiety and executive function; however, additional studies are needed to confirm this. Fourth, we did not find a relationship between anxiety and EEG findings, nor between anxiety and cognitive function. This may be due to the high, clinical level of anxiety among the participants. In this study, only participants who had 40 points or more on either subscale of the STAI were included, and no control group was used. The means of both the STAI (state) and STAI (trait) were very high at 59.63 ± 11.74 and 59.54 ± 10.86, respectively, which may have introduced a bias. Therefore, the methods limited the ability to test the hypothesis that as anxiety levels increase there will be changes in qEEG. Given the limitation of the method, it may be premature to conclude that there is no correlation between severity of anxiety and EEG analyses. Further studies, with controls, are necessary to assess the correlation between anxiety severity and qEEG. Fifth, this study was based on a single local university medical centre. Patients who visited the hospital often complained of severe anxiety at a pathological level. As a result of this study, it is difficult to explain the normal range of anxiety.

The strengths of this study are: first, the study analysed qEEG in patients complaining of anxiety based on the RDoC framework, not on conventional disease-oriented studies. The participants of this study were heterogeneous that can be classified as various mental disorders, but it is more reasonable to interpret the findings from the perspective of the RDoC framework in that anxiety was their chief complaint. Furthermore, the severity of depression capable of affecting the study results was statistically adjusted and significant levels were set using a stricter Bonferroni correction to ensure statistical significance when considering multiple comparison error. In future studies, it is believed that accumulating relevant knowledge using these approaches and statistical methods will be effective in finding pathophysiology and developing novel treatments. Second, all participants in this study were patients who visited the hospital for the first time and had never taken medication. Treatments such as medication^35,36^ or repetitive transcranial magnetic stimulation^37^ were known to alter qEEG results. The results of this study could exclude these factors based on participants who were not affected by the drug.

The purpose of this study was to determine whether the decline in executive function in an adult with anxiety is related to a specific frequency band of qEEG in resting state. This study found that the increase in delta power in the central region and left P3 was negatively correlated with the decrease in executive function. Based on these results, it is expected that the absolute delta power plays a specific role in the task-negative default mode network in the relationship between anxiety and executive function. In contrast to previous studies focused on certain diagnosed mental disorder, this study attempted to uncover the role of specific physiology based on the negative valence domain and potential threat construct of the RDoC framework. To expand the results of this study and broaden the biological understanding of the human mind through the RDoC framework, studies on the association of qEEG indicators and cognitive functions to more diverse psychiatric symptoms will be needed in the future. If this evidence is expanded, it can be used to objectively evaluate the symptoms and treatment responses of patients using quantified neurobiological markers.

## Methods

### Participants

Individuals who visited the psychiatric outpatient clinic of the Daegu Catholic University Hospital from 2017 to 2018 were considered for inclusion in the study. Inclusion criteria constituted individuals with 1) anxiety symptoms as the main complaint and age between 19 and 70 years, and 2) STAI (state) or STAI (trait) score > 40, meaning probable clinical levels of anxiety^38^. Individuals with brain damage, neurological and/or genetic disorders, substance dependence, epilepsy, or any other mental disorder reported during a personal history and anamnesis taking process were excluded from participation. Individuals who had an intelligence quotient (IQ) of 70 or lower according to the Wechsler Adult Intelligence Scale, 4th edition, Korean version (K-WAIS-IV) or who needed immediate intervention due to the possibility of self-harm and aggressive behaviour were also excluded from this study. All participants provided informed consent after being given a complete description of the study and were evaluated for inclusion by an experienced clinical psychiatrist. After being enrolled, participants visited the clinic again and performed the EEG recoding, IQ test, and Executive Intelligence Test (EXIT) composed of the Stroop, verbal fluency, and design fluency tests on the same day. Participants also completed demographic and clinical questionnaires including the STAI (both state and trait) and BDI on the same day. This study was approved by the Institutional Review Board (IRB) of the Daegu Catholic University Medical Centre (DCUMC IRB approval No. CR-19–005) and was performed in accordance with the Declaration of Helsinki (World Medi-cal Association: Ethical Principles for Medical Research In-volving Human Subjects, 1964).

### Anxiety symptoms: State-Trait Anxiety Inventory (STAI)

STAI is a tool designed to evaluate anxiety in terms of ‘here and now’ (state) and in general (trait), and has proven useful in measuring anxiety in the general population and clinical samples^39^. Each subscale has 20 items answered on a 4-point Likert scale and scores above 40 are usually defined as clinical anxiety^38^. This measure was used to select participants, excluding those with scores < 40 on either subscale.

### Depressive symptoms: Beck Depression Inventory (BDI)

The BDI is the most popular self-rating scale and it was developed by Beck et al.^40^ It includes a 4-point Likert scale of 0 to 3 points for a total score range of 0 to 63, calculated by 21 items. It has been translated into Korean by Hahn et al.^41^ According to Hahn et al., the reliability coefficient of the Korean version of the BDI was 0.886. We used this scale to evaluate depressive symptoms among participants as their depressive symptoms could be associated with anxiety. We adjusted the BDI for evaluating anxiety.

### Executive Intelligence Test (EXIT)

We used Kims Executive Intelligence Test (EXIT)^42^ as an objective and quantitative tool for measuring executive function. It consists of three tests to assess domains of participants’ executive function including concentration, vocabulary, visuospatial ability, and memory. The result of EXIT is calculated as the EIQ, of which the mean is 100 and standard deviation is 15.

### Inhibition: Stroop test

The Stroop test consists of three trials: a word, a colour, and a word-colour interference trial. This study used the translated Korean version^42^. In the word-naming trial, the participants were told to read aloud four-colour words printed in black. In the colour-naming trial, the words were printed in the colour to which they referred (i.e., word and colour were congruent), and the participants were told to read the words aloud. Finally, in the colour-word interference trial, the words were incongruent with their ink colour, and the participants were instructed to say the name of the ink colour and not read the word. In this study, the results were presented in terms of response times (i.e., the time between the presentation of the target stimulus and the response).

### Verbal fluency: Controlled Oral Word Association Test

The Controlled Oral Word Association Test (COWAT) is a standardised version of a word fluency test^43^. It measures phonemic and semantic fluencies. In the phonemic fluency test, participants were told to list as many words as they could think of that begin with the three Korean letters that resemble the letters "g," "o," and "s" during three separate 1-min trials. In the semantic fluency test, participants were instructed to provide examples of fruit and animal categories during two separate 1-min trials. In this study, the sum of the phonemic and semantic fluency scores was used to indicate verbal fluency.

### Design fluency test

The design fluency test that we used was a Korean standardised version of the Ruff Figural Fluency Test^44^. All participants were instructed to connect five separate points in as many ways as possible in 1 min. Total scores were calculated based on the number of different designs made in three trials.

### EEG recording and pre-processing

This study's methods (‘EEG recording, pre-processing, power-spectrum analysis, and statistical analysis’) were the same as the core methodology used in the authors' previous studies^45–49^. In the past few years, the authors have studied the use of qEEG as a diagnostic marker for psychiatric disorders as described in the DSM-5, such as attention-deficit hyperactivity disorder and schizophrenia, using the same research methodology as in this study. Here, the existing EEG protocol was used to study the relationship between qEEG and executive function for anxiety symptoms in the RDoC system.

The EEG recordings were performed using a SynAmps2 direct-current (DC) amplifier and a 10–20 layout 64-channel Quick-Cap electrode placement system (Neuroscan Inc., NC, USA). The EEG data were digitally recorded from 21 gold cup electrodes that were placed according to the International 10–20 system. The impedances were maintained below 5 kΩ, and the sampling rate was 1000 Hz. We used the linked mastoid reference and two additional bipolar electrodes to measure both the horizontal and vertical eye movements. During the recording, each participant laid in a semi-darkened, electrically shielded, sound-attenuated room and were instructed to relax and avoid any body movements or drowsiness to reach the resting state. The resting state EEG recording was conducted for 15 min under the following conditions to prevent the brain waves from changing after falling asleep: 5 min with eyes closed (EC), 5 min with eyes open (EO), and 5 min with EC. We analysed the EO EEG recordings as EO has been suggested to be more suitable when assessing visual tasks than EC^50^.

We used Matlab 7.0.1 (Math Works, Natick, MA, USA) and the EEGLAB toolbox^51^ to pre-process and analyse the EEG recordings. First, the EEG data were downsampled to 250 Hz and were subsequently detrended and mean-subtracted to remove the DC component. A 1-Hz high-pass filter and a 60-Hz notch filter were applied to remove the eye and electrical noises. Moreover, an independent component analysis (ICA) was performed to remove the well-defined sources of artefacts. ICA has been demonstrated to reliably isolate artefacts caused by eye and muscle movements and heart noise^52^. Components that corresponded to eye blinking or muscle movement were identified using a published technique that is superior to other artefact rejection techniques^53^. We identified and removed at least one component that corresponded to muscle artefacts, and no detected residual muscle artefacts. Finally, clinical psychiatrists and EEG experts visually inspected the corrected EEGs for noisy epochs and channels. Deleted channels were interpolated and recordings were re-referenced to a common average reference. For the analysis, we selected more than 3 min of EO artefact-free EEG readings from the 5-min recordings.

### Power-spectrum analysis of the EEG recordings

Five frequency bands were defined for further analysis: delta (1–4 Hz), theta (4–8 Hz), slow alpha (8–10 Hz), fast alpha (10–13.5 Hz), and beta (13.5–30 Hz). The spectral power of the EEG data was calculated via a fast Fourier transformation using the “spectrogram.m” function of the signal processing toolbox in Matlab. Time windows of 1000 ms with an 800 ms overlap and the Hamming window were used for the spectral analysis. Finally, the absolute powers were averaged over all time windows and frequency bands for further analysis.

#### Statistical analyses

All values are expressed as the mean and standard deviation (SD). To assess the relationship between the cognitive function and EEG recordings, we used Pearson’s partial correlation analysis that controlled for age, sex, and BDI. Statistical significance was defined as *p* < 0.0026 (0.05/19). To control for false positives from multiple comparisons, we used the Bonferroni correction in which the *p* values were multiplied by the number of comparisons for 19 electrodes, placed according to the International 10–20 system. To improve clarity, topographical plots of the results of Pearson’s partial correlations are presented. In addition, Pearson's partial correction analysis that controlled for age, gender, and BDI, were used to analyse the association between anxiety and the EEG recordings, and anxiety and cognitive function. For analyses of anxiety and EEG recordings, statistical significance was *p* < 0.0026 (0.05/19). For anxiety and cognitive function analyses, statistical significance was corrected based on the number of EXIT tests (i.e., 6), thus *p* < 0.083 (0.05/6). All data were analysed using the Statistical Package for the Social Sciences (SPSS) software, version 18.0 (SPSS Inc., Chicago, IL, USA).

## Supplementary information

Supplementary Information

## Data Availability

The full dataset used in this study can be available from the corresponding author on reasonable request.
