# Supplementary material for: Correlation between executive function and quantitative EEG in patients with anxiety by the Research Domain Criteria (RDoC) framework
Source: Sci Rep. 2020 Oct 29;10:18578. doi: 10.1038/s41598-020-75626-0 (PMC7596478; doi:10.1038/s41598-020-75626-0)
Supplement: Supplementary file 1 — Supplementary Information [file 41598_2020_75626_MOESM1_ESM.docx]

**Correlation between executive function and quantitative EEG in patients with anxiety by the Research Domain Criteria (RDoC) framework**

**Authors:** Su Hyun Bong^a^, Tae Young Choi^a^, Kyoung Min Kim^b^, Jaewon Lee^c^, Jun Won Kim^a,*^

^a^Department of Psychiatry, Catholic University of Daegu School of Medicine, Daegu, Republic of Korea

^b^Department of Psychiatry, College of Medicine, Dankook University, Cheonan, Republic of Korea

^c^Department of Psychiatry, Easybrain Centre, Seoul, Republic of Korea

**^*^Corresponding author:**

Jun Won Kim

**Affiliation:** Department of Psychiatry, Catholic University of Daegu School of Medicine, Daegu, Republic of Korea

**Address:** Department of Psychiatry, Catholic University of Daegu School of Medicine, 33, Duryugongwon-ro 17-gil, Nam-gu, Daegu, 42471, Republic of Korea

**Email address:** [f_affection@naver.com](mailto:f_affection@naver.com)

**Phone number**: +82-53-650-4780

**Fax number**: +82-53-623-7507

S1. The results of the Pearson’s partial correlation analysis (corrected for age, gender and BDI) between STAI (state) and EEG analysis

| *STAI (state)* | | Delta | Theta | Slow alpha | Fast alpha | Beta |
| --- | --- | --- | --- | --- | --- | --- |
| Fp1 | *R* | *-0.023* | *-0.132* | *-0.245* | *-0.169* | *0.030* |
|  | *P_Bon_* | *0.892* | *0.429* | *0.139* | *0.312* | *0.857* |
| Fp2 | *R* | *0.085* | *-0.017* | *-0.194* | *-0.165* | *0.152* |
|  | *P_Bon_* | *0.614* | *0.919* | *0.243* | *0.323* | *0.361* |
| F7 | *R* | *0.154* | *0.073* | *-0.099* | *-0.049* | *0.216* |
|  | *P_Bon_* | *0.356* | *0.665* | *0.556* | *0.770* | *0.192* |
| F3 | *R* | *0.015* | *-0.120* | *-0.118* | *-0.024* | *0.139* |
|  | *P_Bon_* | *0.928* | *0.471* | *0.479* | *0.887* | *0.406* |
| Fz | *R* | *0.234* | *-0.003* | *-0.117* | *-0.026* | *0.202* |
|  | *P_Bon_* | *0.156* | *0.984* | *0.484* | *0.879* | *0.223* |
| F4 | *R* | *-0.050* | *-0.056* | *-0.107* | *-0.018* | *0.208* |
|  | *P_Bon_* | *0.766* | *0.740* | *0.522* | *0.913* | *0.211* |
| F8 | *R* | *-0.190* | *-0.129* | *-0.164* | *-0.137* | *0.139* |
|  | *P_Bon_* | *0.254* | *0.441* | *0.326* | *0.411* | *0.407* |
| T7 | *R* | *-0.055* | *0.004* | *-0.066* | *-0.026* | *0.164* |
|  | *P_Bon_* | *0.743* | *0.983* | *0.695* | *0.876* | *0.324* |
| C3 | *R* | *0.037* | *0.044* | *-0.003* | *-0.037* | *0.175* |
|  | *P_Bon_* | *0.823* | *0.795* | *0.988* | *0.824* | *0.294* |
| Cz | *R* | *0.004* | *0.001* | *-0.090* | *-0.024* | *0.196* |
|  | *P_Bon_* | *0.981* | *0.994* | *0.590* | *0.884* | *0.239* |
| C4 | *R* | *0.193* | *-0.043* | *-0.129* | *-0.143* | *0.101* |
|  | *P_Bon_* | *0.245* | *0.797* | *0.441* | *0.392* | *0.548* |
| T8 | *R* | *-0.128* | *-0.143* | *-0.099* | *-0.144* | *0.032* |
|  | *P_Bon_* | *0.444* | *0.392* | *0.555* | *0.387* | *0.847* |
| P7 | *R* | *0.052* | *-0.069* | *-0.128* | *-0.032* | *0.177* |
|  | *P_Bon_* | *0.755* | *0.679* | *0.445* | *0.849* | *0.287* |
| P3 | *R* | *0.265* | *0.137* | *0.021* | *0.014* | *0.205* |
|  | *P_Bon_* | *0.108* | *0.414* | *0.903* | *0.931* | *0.216* |
| Pz | *R* | *0.174* | *0.055* | *-0.114* | *-0.043* | *0.126* |
|  | *P_Bon_* | *0.296* | *0.742* | *0.494* | *0.796* | *0.450* |
| P4 | *R* | *0.126* | *-0.087* | *-0.130* | *-0.101* | *0.086* |
|  | *P_Bon_* | *0.451* | *0.604* | *0.438* | *0.546* | *0.607* |
| P8 | *R* | *-0.032* | *-0.155* | *-0.130* | *-0.106* | *0.119* |
|  | *P_Bon_* | *0.850* | *0.354* | *0.436* | *0.527* | *0.477* |
| O1 | *R* | *0.237* | *0.088* | *-0.135* | *-0.023* | *0.226* |
|  | *P_Bon_* | *0.153* | *0.601* | *0.420* | *0.893* | *0.172* |
| O2 | *R* | *0.233* | *0.040* | *-0.177* | *-0.098* | *0.185* |
|  | *P_Bon_* | *0.160* | *0.812* | *0.288* | *0.557* | *0.266* |

* *p*<0.0026(0.05/19), R means Pearson’s partial correlation coefficient; P means p-value of Pearson’s partial correlation; P_Bon_ means the p-value adjusted using the Bonferroni correction; STAI (state), State-Trait Anxiety Inventory (state); BDI, Beck Depression Inventory; EEG, electroencephalogram

S2. The results of the Pearson’s partial correlation analysis (corrected for age, gender and BDI) between STAI (trait) and EEG analysis

| *STAI (trait)* | | Delta | Theta | Slow alpha | Fast alpha | Beta |
| --- | --- | --- | --- | --- | --- | --- |
| Fp1 | *R* | *-0.109* | *-0.142* | *-0.103* | *-0.043* | *-0.063* |
|  | *P_Bon_* | *0.514* | *0.396* | *0.537* | *0.798* | *0.706* |
| Fp2 | *R* | *0.094* | *0.065* | *-0.015* | *-0.030* | *0.146* |
|  | *P_Bon_* | *0.574* | *0.699* | *0.928* | *0.858* | *0.382* |
| F7 | *R* | *0.121* | *0.070* | *0.060* | *0.175* | *0.169* |
|  | *P_Bon_* | *0.471* | *0.677* | *0.721* | *0.292* | *0.312* |
| F3 | *R* | *-0.070* | *-0.115* | *-0.034* | *0.089* | *0.057* |
|  | *P_Bon_* | *0.676* | *0.492* | *0.841* | *0.594* | *0.736* |
| Fz | *R* | *-0.055* | *-0.109* | *-0.050* | *0.070* | *0.060* |
|  | *P_Bon_* | *0.743* | *0.515* | *0.766* | *0.674* | *0.720* |
| F4 | *R* | *-0.244* | *-0.089* | *-0.027* | *0.068* | *0.057* |
|  | *P_Bon_* | *0.139* | *0.593* | *0.870* | *0.686* | *0.735* |
| F8 | *R* | *-0.095* | *-0.062* | *0.026* | *0.078* | *0.104* |
|  | *P_Bon_* | *0.572* | *0.712* | *0.879* | *0.643* | *0.536* |
| T7 | *R* | *-0.033* | *0.018* | *0.034* | *0.161* | *0.053* |
|  | *P_Bon_* | *0.845* | *0.914* | *0.841* | *0.333* | *0.750* |
| C3 | *R* | *-0.154* | *-0.116* | *-0.048* | *-0.051* | *-0.001* |
|  | *P_Bon_* | *0.357* | *0.489* | *0.774* | *0.762* | *0.994* |
| Cz | *R* | *-0.152* | *-0.131* | *-0.087* | *0.043* | *0.036* |
|  | *P_Bon_* | *0.362* | *0.434* | *0.602* | *0.796* | *0.832* |
| C4 | *R* | *-0.094* | *-0.123* | *-0.051* | *-0.037* | *-0.019* |
|  | *P_Bon_* | *0.575* | *0.464* | *0.759* | *0.825* | *0.909* |
| T8 | *R* | *-0.123* | *-0.109* | *-0.008* | *0.047* | *-0.003* |
|  | *P_Bon_* | *0.463* | *0.515* | *0.960* | *0.780* | *0.988* |
| P7 | *R* | *-0.031* | *-0.037* | *0.044* | *0.167* | *0.075* |
|  | *P_Bon_* | *0.851* | *0.827* | *0.791* | *0.317* | *0.656* |
| P3 | *R* | *0.053* | *0.003* | *0.091* | *0.075* | *0.062* |
|  | *P_Bon_* | *0.753* | *0.986* | *0.587* | *0.656* | *0.711* |
| Pz | *R* | *0.008* | *-0.096* | *-0.031* | *0.033* | *0.015* |
|  | *P_Bon_* | *0.964* | *0.565* | *0.853* | *0.843* | *0.928* |
| P4 | *R* | *-0.006* | *-0.125* | *-0.018* | *0.020* | *0.012* |
|  | *P_Bon_* | *0.973* | *0.454* | *0.913* | *0.906* | *0.944* |
| P8 | *R* | *-0.184* | *-0.199* | *-0.084* | *-0.069* | *-0.055* |
|  | *P_Bon_* | *0.270* | *0.230* | *0.617* | *0.680* | *0.744* |
| O1 | *R* | *-0.011* | *-0.020* | *-0.035* | *0.066* | *0.072* |
|  | *P_Bon_* | *0.948* | *0.904* | *0.835* | *0.693* | *0.665* |
| O2 | *R* | *-0.055* | *-0.133* | *-0.152* | *-0.080* | *0.023* |
|  | *P_Bon_* | *0.742* | *0.425* | *0.362* | *0.635* | *0.893* |

* *p*<0.0026(0.05/19), R means Pearson’s partial correlation coefficient; P means p-value of Pearson’s partial correlation; P_Bon_ means the p-value adjusted using the Bonferroni correction; STAI (trait), State-Trait Anxiety Inventory (trait); BDI, Beck Depression Inventory; EEG, electroencephalogram

S3. The results of the Pearson’s partial correlation analysis (corrected for age, gender and BDI) between STAI and cognitive function

| *STAI* | | EIQ |  | Stroop |  | Verbal | Design |
| --- | --- | --- | --- | --- | --- | --- | --- |
|  |  |  | Simple | Midterm | Interference |  |  |
| STAI (state) | *R* | *-0.162* | *-0.013* | *0.004* | *0.032* | *-0.141* | *-0.218* |
|  | *P_Bon_* | *0.332* | *0.940* | *0.980* | *0.847* | *0.398* | *0.189* |
| STAI (trait) | *R* | *0.041* | *-0.002* | *0.013* | *0.265* | *-0.049* | *0.078* |
|  | *P_Bon_* | *0.808* | *0.990* | *0.938* | *0.108* | *0.769* | *0.641* |

* p<0.0083(0.05/6), R means Pearson’s partial correlation coefficient; P means p-value of Pearson’s partial correlation; P_Bon_ means the p-value adjusted using the Bonferroni correction; STAI (state), State-Trait Anxiety Inventory (state); STAI (trait), State-Trait Anxiety Inventory (trait); BDI, Beck Depression Inventory; EIQ, Executive Intelligence Quotient
